# Correction: Risk factors and pharmacotherapy for chemotherapy-induced peripheral neuropathy in paclitaxel-treated female cancer survivors: A retrospective study in Japan

**DOI:** 10.1371/journal.pone.0307865

**Published:** 2024-07-23

**Authors:** Shiori Hiramoto, Hajime Asano, Tomoyoshi Miyamoto, Manabu Takegami, Atsufumi Kawabata

In Table 2, the data for Multivariate columns in BMI are missing. Please see the correct Table 2 here.

**Table 2:** Association of various factors with diagnosis of PIPN in female cancer patients.

	Diagnosis of PIPN			
	%		Multivariate	
Factor	(Yes/Yes+No)	*p*	Odds ratio (95% CI)	*p*
Cancer type				
Breast cancer	84.6 (137/162)			
Gynecologic cancer	83.5 (101/121)	0.870		
Age (Median: 58)				
< 58	79.6 (109/137)		Reference	
≥ 58	88.4 (129/146)	0.0511	2.03 (1.03–4.00)	**0.040**
Age (Optimal cutoff value: 55)				
< 55	77.8 (91/117)			
≥ 55	88.6 (147/166)	**0.0202**		
Total dose of PCT (Median: 944.9)				
< 944.9 mg/m^2^	78.0 (110/141)		Reference	
≥ 944.9 mg/m^2^	90.1 (128/142)	**0.00575**	2.83 (1.35–5.91)	**0.00575**
Total dose of PCT (Optimal cutoff value: 924.8)				
< 924.8 mg/m^2^	74.6 (85/114)			
≥ 924.8 mg/m^2^	90.5 (153/169)	**0.000439**		
Addition of platinum agents				
No	84.6 (137/162)		Reference	
Yes	83.5 (101/121)	0.870	0.669 (0.331–1.35)	0.261
Surgery				
No	86.7 (13/15)			
Yes	84.0 (225/268)	1.00		
Metastasis				
No	86.7 (117/135)			
Yes	81.8 (121/148)	0.329		
Medical history of female hormone-related diseases				
No	82.0 (205/250)			
Yes	100 (33/33)	**0.00403**		
Diabetes				
No	83.3 (224/269)			
Yes	100 (14/14)	0.137		
Hyperlipidemia				
No	84.0 (226/269)			
Yes	85.7 (12/14)	1.00		
Hypertension				
No	81.8 (189/231)			
Yes	94.2 (49/52)	**0.0338**		
BSA (Median: 1.526)				
< 1.526	82.3 (116/141)			
≥ 1.526	85.9 (122/142)	0.421		
BMI (Median: 21.8)				
< 21.8	78.2 (111/142)		Reference	
≥ 21.8	90.1 (127/141)	**0.00878**	2.2 (1.09–4.41)	**0.0267**

The association between various factors and PIPN diagnosis was analyzed in a dichotomous manner, where continuous variables were divided into two categories using medians. The optimal cutoff values for patients’ age and total dose of paclitaxel (PCT) were also determined from the receiver operating characteristic (ROC) curves, and used for categorization. BSA, body surface area; BMI, body mass index. Effect of each factor on PIPN diagnosis was analyzed by Fisher’s exact test. Further, effects of age, total dose of PCT, BMI and addition of platinum agents on PIPN diagnosis were evaluated by multivariate logistic regression analysis. CI, confidence interval.

In the Statistical Analysis subsection of Methods, there is an error in the fifth sentence. The correct sentence is: Multivariate logistic regression analysis was conducted to test the association of age, total dose of paclitaxel, BMI and addition of platinum agents with PIPN diagnosis, showing odds ratios with 95% confidence intervals (CIs).

In Fig 1, the *p* values are incorrect. Please see the correct Fig 1 here.

**Fig 1:**
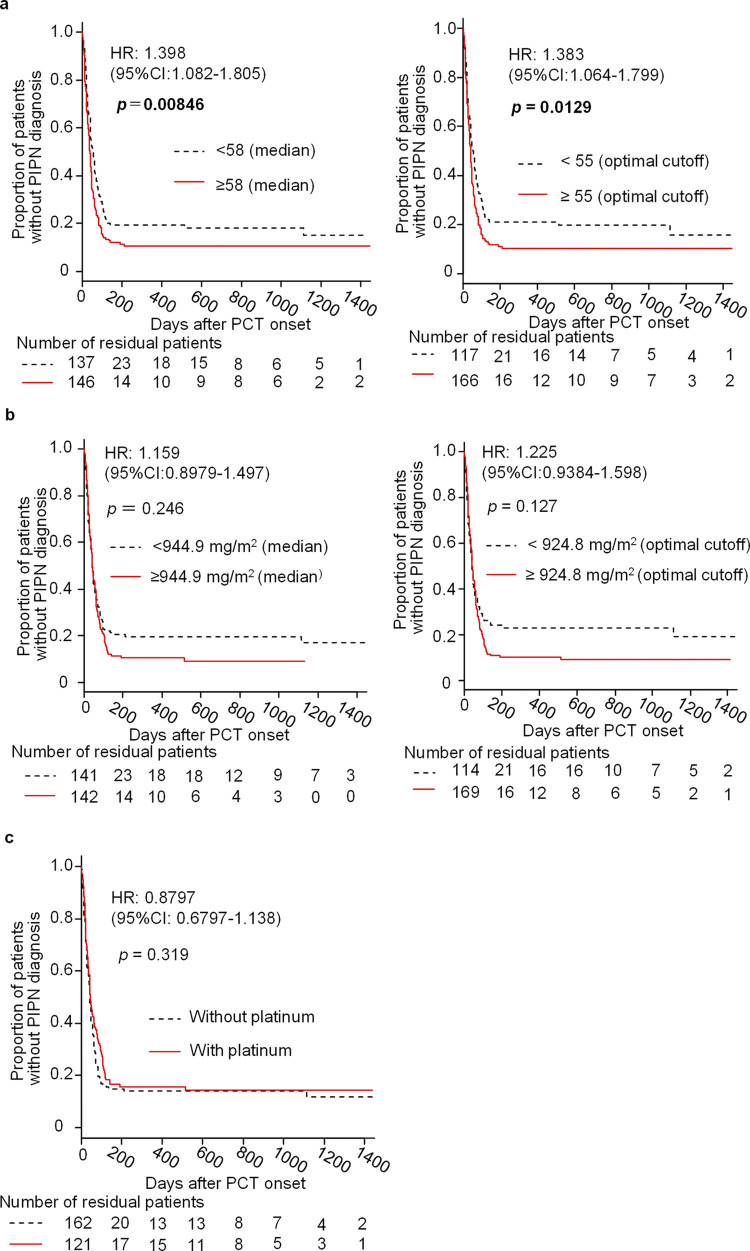
Kaplan-Meier curves for the time-related increase in PIPN diagnosis in female cancer patients treated with paclitaxel (PCT), and their association with older age (a), greater total paclitaxel dose (b) and additional administration of platinum agents (c). Medians were 58 years of age (a, left) and 944.9 mg/m2 of total paclitaxel dose (b, left), and optimal cutoff values estimated by ROC analysis were 55 (a, right) and 924.8 (b, right), respectively. Statistical significance between two groups was analyzed by a log-rank test. Hazard ratios (HRs) with 95% CIs were calculated using a Cox proportional hazard regression model.

In the Association of various factors with diagnosis of PIPN in female cancer survivors who underwent paclitaxel-based chemotherapy subsection of Results, there is an error in the second and third sentences of the second paragraph. The correct sentences are: The log-rank test indicated significant impact of age ≥ 58 (median) and ≥ 55 (optimal cut-off value) years on PIPN diagnosis (p = 0.00846 and 0.0129, respectively), and the Cox proportional hazard model provided hazard ratios, 1.398 (95%CI, 1.082–1.805) and 1.383 (95%CI, 1.064–1.799), respectively (Fig 1A). On the other hand, total paclitaxel dose, regardless using medians or optical cut-off values for categorization, or additional platinum agents had no significant impact on PIPN diagnosis (Fig 1B and 1C).
