# Supplementary material for: Risk factors and pharmacotherapy for chemotherapy-induced peripheral neuropathy in paclitaxel-treated female cancer survivors: A retrospective study in Japan
Source: PLoS One. 2021 Dec 31;16(12):e0261473. doi: 10.1371/journal.pone.0261473 (PMC8719717; doi:10.1371/journal.pone.0261473)
Supplement: S1 Fig — PCT, paclitaxel. *Two patients had both ovarian and fallopian tube cancers. (PDF) [file pone.0261473.s001.pdf]

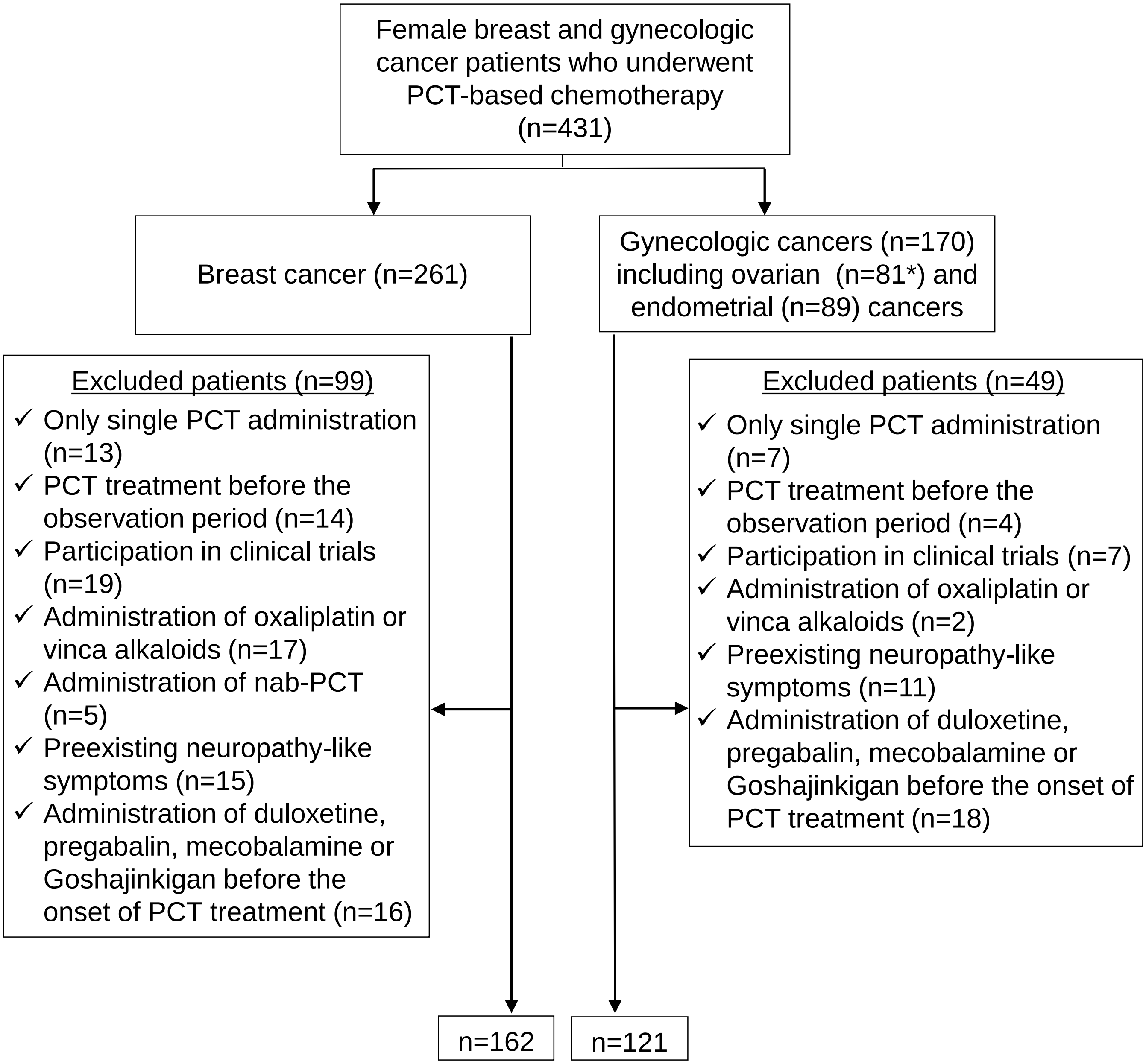

S1 Fig. Diagram of patient selection. PCT, paclitaxel. *Two patients had both ovarian and fallopian tube cancers.
