# Supplementary material for: Risk factors and pharmacotherapy for chemotherapy-induced peripheral neuropathy in paclitaxel-treated female cancer survivors: A retrospective study in Japan
Source: PLoS One. 2021 Dec 31;16(12):e0261473. doi: 10.1371/journal.pone.0261473 (PMC8719717; doi:10.1371/journal.pone.0261473)
Supplement: S2 Fig — The top graphs show box-and-whisker plots. Statistical significance was analyzed by Mann-Whitney’s U test. (PDF) [file pone.0261473.s002.pdf]

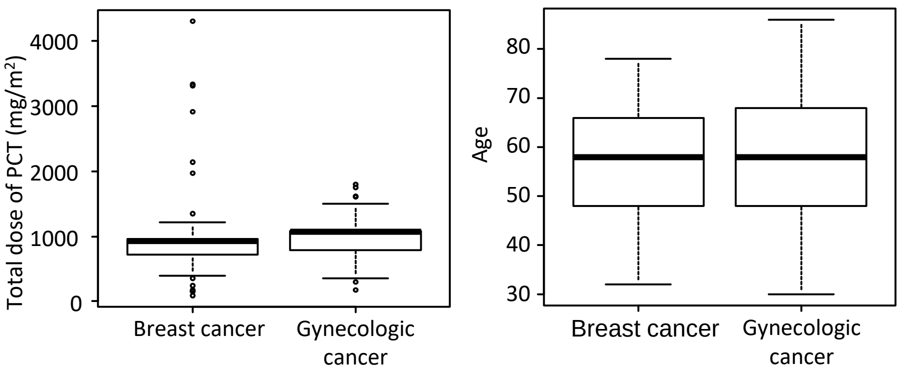

	Breast cancer (n=162)	Gynecologic cancer (n=121)	*p*
Total dose of PCT (mg/m^2), median [range]	931.1 [89.6, 4311.4]	1072.2 [178.5,1759.5]	**<0.0001**
Age, median [range]	58 [32,78]	58 [30,86]	0.833

S2 Fig. Comparison of total dose of paclitaxel (PCT) and age between breast and gynecologic cancer patients. The top graphs show box-and-whisker plots. Statistical significance was analyzed by Mann-Whitney's U test.
