# Supplementary material for: Risk factors and pharmacotherapy for chemotherapy-induced peripheral neuropathy in paclitaxel-treated female cancer survivors: A retrospective study in Japan
Source: PLoS One. 2021 Dec 31;16(12):e0261473. doi: 10.1371/journal.pone.0261473 (PMC8719717; doi:10.1371/journal.pone.0261473)
Supplement: S1 File — (PDF) [file pone.0261473.s003.pdf]

ID after anonymization	cancer type	ope	age	hormone diseases			DM	HL	HT	BSA	BMI	NP	VB12	Goshajinkigan	duloxetine	pregabalir	regimen	with Pt	total dose	period of diagnosis	metastasis
				ovarian cyst	uterine fiobroid	endmetriosis															
1259	breast	1	61	0	0	0	0	0	0	1.485	23.7	1	0	0	0	1	wPTX+HER	0	960.00	49	1
3523	gynecology	1	61	1	0	0	1	0	0	1.572	24.5	1	0	0	0	0	tri-weekly TC	1	1076.34	34	1
5168	breast	1	42	0	0	0	0	0	0	1.436	19.6	1	0	0	0	0	wPTX+HER	0	952.65	63	1
11116	breast	1	43	0	0	0	0	0	0	1.588	24.7	1	0	0	0	1	wPTX	0	944.58	21	1
11738	gynecology	1	53	0	0	0	1	0	0	1.832	26.3	1	0	0	0	1	tri-weekly TC	1	553.65	16	0
17324	breast	1	73	0	0	0	0	0	0	1.333	18.6	0	0	0	0	0	ddPTX	0	690.17	634	1
21991	breast	1	68	0	0	0	0	0	1	1.423	20.0	1	0	0	1	1	wPTX	0	720.00	28	0
23456	gynecology	1	67	0	0	0	0	0	0	1.549	23.9	1	1	0	0	1	tri-weekly TC	1	941.08	98	1
27171	gynecology	0	45	0	0	0	0	0	0	1.578	20.6	1	0	0	0	0	tri-weekly TC+BEV	1	1007.60	28	1
29175	gynecology	1	41	0	0	0	0	0	0	1.636	13.3	0	1	0	0	0	tri-weekly TC	1	718.98	1300	0
29428	breast	1	69	0	0	0	0	0	0	1.266	17.1	1	0	0	0	0	wPTX	0	960.00	28	0
35055	gynecology	1	54	0	0	0	0	0	1	1.557	20.1	1	0	0	0	0	tri-weekly TC+BEV	1	927.73	41	0
40484	breast	1	36	0	0	0	0	0	0	1.435	19.2	0	0	0	0	0	wPTX	0	953.31	237	0
46620	breast	1	47	0	0	0	0	0	0	1.701	26.2	1	0	0	0	0	wPTX	0	399.76	14	1
47482	breast	1	49	0	0	0	0	0	0	1.671	25.0	1	0	0	0	1	wPTX	0	933.57	14	1
47671	gynecology	1	51	0	0	0	0	0	1	1.524	23.6	1	0	0	1	1	tri-weekly TC	1	1078.74	20	0
58734	gynecology	1	53	0	0	0	0	0	0	1.407	20.7	0	0	0	0	0	tri-weekly TC	1	355.37	27	0
62180	gynecology	1	71	0	0	0	0	0	0	1.522	18.2	1	1	1	0	0	tri-weekly TC	1	538.11	3	0
63534	gynecology	1	66	1	0	0	0	0	0	1.551	24.2	1	1	1	0	0	tri-weekly TC	1	1079.30	42	1
73808	gynecology	1	57	0	0	0	0	0	0	1.557	22.3	0	0	0	0	0	tri-weekly TC+BEV	1	1069.01	417	1
74206	breast	0	58	0	0	0	0	0	0	1.366	18.2	1	0	0	0	0	wPTX+BEV	0	3338.21	39	1
75004	breast	1	70	0	0	0	0	0	1	1.397	21.8	1	0	0	0	0	wPTX+HER	0	944.88	67	0
84643	breast	1	54	0	0	0	0	0	0	1.689	26.3	1	0	1	1	1	wPTX	0	959.15	63	0
86089	breast	1	47	0	1	0	0	0	0	1.514	21.2	1	0	0	0	0	wPTX	0	951.12	21	0
102834	breast	1	45	0	0	0	0	0	0	1.444	18.4	1	0	0	0	0	wPTX	0	955.68	7	0
103278	gynecology	1	73	0	0	0	0	1	1	1.454	17.8	1	0	0	0	0	tri-weekly TC	1	1074.28	64	1
106543	gynecology	1	39	0	0	0	0	1	0	1.678	25.5	0	0	0	0	0	tri-weekly TC	1	475.17	915	0
108692	breast	1	76	0	0	0	0	0	0	1.234	15.1	1	0	0	0	0	wPTX	0	766.61	98	1
120053	gynecology	1	60	0	0	0	1	0	0	1.53	22.6	1	1	1	1	1	tri-weekly TC	1	1258.17	11	1
121238	breast	1	63	0	0	0	0	0	0	1.309	14.8	0	0	0	0	0	wPTX+HER	0	916.73	889	1
125853	gynecology	1	45	0	0	0	0	0	0	1.325	15.7	1	0	0	0	0	tri-weekly TC+BEV	1	1052.40	87	1
126860	gynecology	1	66	0	0	0	0	0	1	1.534	30.7	1	1	1	0	1	tri-weekly TC	1	1501.27	20	1
135952	gynecology	1	51	0	0	0	0	0	1	1.526	329.8	0	0	0	0	0	tri-weekly TC	1	898.89	638	0
138916	breast	1	45	0	1	0	0	0	0	1.389	19.4	1	0	1	0	1	wPTX	0	879.05	21	1

139167	gynecology	1	70	0	0	0	0	0	0	1.537	25.2	0	0	0	0	0	tri-weekly TC	1	1041.44	329	1
144833	breast	1	68	0	0	0	0	0	0	1.389	19.9	1	0	0	0	0	wPTX+HER	0	871.13	70	1
146930	gynecology	1	86	0	0	0	0	0	1	1.293	20.1	1	0	0	0	0	weekly TC	1	893.27	140	0
146958	gynecology	1	62	0	0	0	0	0	1	1.536	24.3	0	0	0	0	0	tri-weekly TC	1	1078.13	1131	1
148644	breast	1	65	0	0	0	1	0	1	1.605	28.1	1	0	0	0	0	ddPTX	0	697.82	14	0
148736	gynecology	1	69	0	0	0	0	0	0	1.413	23.9	1	1	1	0	0	tri-weekly TC	1	700.75	63	0
148764	gynecology	1	55	0	0	0	0	0	0	1.573	19.1	1	0	0	0	0	tri-weekly TC	1	893.90	107	0
151282	breast	1	48	0	0	0	0	0	0	1.514	19.4	1	0	1	0	0	ddPTX	0	673.71	0	0
151323	gynecology	1	70	0	0	0	1	0	0	1.542	23.6	1	0	0	1	0	tri-weekly TC	1	1077.82	3	1
153123	gynecology	1	39	0	0	0	0	0	1	1.678	25.5	0	0	0	0	0	tri-weekly TC	1	475.17	915	0
155036	breast	1	52	0	0	0	0	0	0	1.696	26.5	1	0	0	0	1	wPTX	0	955.19	63	0
158470	gynecology	1	42	0	0	0	0	0	0	1.498	21.4	0	0	0	0	0	tri-weekly TC	1	1078.95	670	1
159779	gynecology	1	76	0	0	0	0	0	0	1.48	19.1	1	0	0	0	0	tri-weekly TC	1	431.76	20	0
163580	breast	1	67	0	0	0	0	0	0	1.323	22.3	1	0	0	0	0	wPTX+BEV	0	952.38	21	0
163964	breast	1	42	0	0	0	0	0	0	1.538	19.3	0	0	0	0	0	ddPTX	0	689.21	656	0
164831	gynecology	1	86	0	0	0	0	0	0	146	7.4	0	0	0	0	0	tri-weekly TC	1	611.37	212	1
165509	gynecology	1	65	0	0	0	0	0	0	1.566	18.0	1	1	1	0	0	tri-weekly TC	1	538.31	32	0
166354	gynecology	1	42	0	0	0	0	0	0	1.265	16.5	1	0	0	0	0	tri-weekly TC	1	1077.40	140	1
166685	breast	1	72	0	0	0	0	0	0	1.444	25.4	1	0	0	0	1	wPTX+HER	0	955.68	63	0
169780	gynecology	1	76	0	0	0	0	0	1	1.616	31.0	1	1	0	0	1	tri-weekly TC	1	1076.73	13	1
170062	breast	1	40	0	0	0	0	0	0	1.728	27.0	1	1	0	1	1	wPTX+HER	0	937.50	72	0
170144	breast	1	64	0	0	0	0	1	1	1.581	27.3	1	0	0	0	0	wPTX	0	956.36	14	0
171285	breast	1	77	0	0	0	0	0	1	1.285	21.5	1	0	0	0	0	wPTX+HER	0	873.15	21	0
172495	gynecology	1	45	0	0	0	0	0	0	1.57	28.6	1	0	0	0	0	tri-weekly TC	1	1075.98	47	1
173816	gynecology	1	48	0	0	0	0	0	0	1.187	15.7	0	0	0	0	0	tri-weekly TC	1	1076.79	708	1
178606	breast	1	70	0	0	0	1	1	1	1.52	23.4	1	0	1	0	1	wPTX+HER	0	789.47	35	0
181651	breast	1	72	0	0	0	0	0	0	1.497	24.1	1	0	0	0	0	wPTX+HER	0	921.84	210	0
186837	breast	1	67	0	0	0	0	0	0	1.509	23.4	1	0	0	0	0	wPTX+HER	0	954.27	21	0
189731	breast	1	52	0	0	0	0	0	0	1.461	18.6	1	0	0	0	0	wPTX	0	157.43	7	1
190369	breast	1	40	0	0	0	0	0	0	1.579	23.9	1	0	0	0	0	wPTX+HER	0	949.97	42	1
191827	breast	1	66	0	0	0	0	0	0	1.456	22.2	1	0	0	0	1	wPTX	0	960.00	63	1
195734	breast	1	53	0	1	0	0	0	0	1.495	0.6	1	0	0	0	0	wPTX	0	955.18	42	1
199822	gynecology	1	51	0	0	0	0	0	0	1.356	21.1	1	0	0	0	0	tri-weekly TC	1	1604.09	114	1
199830	gynecology	1	75	0	0	0	0	0	1	1.526	20.3	1	0	1	0	0	tri-weekly TC	1	1078.07	15	0
200689	breast	1	56	0	0	0	0	0	0	1.455	20.8	1	0	0	0	0	wPTX+HER	0	948.45	28	0
202112	breast	1	51	0	0	0	0	0	0	1.467	18.3	1	0	0	0	0	wPTX	0	899.80	42	1
209568	gynecology	1	60	0	0	0	0	0	1	1.53	28.1	1	1	1	0	0	tri-weekly TC	1	1617.65	22	1
210466	gynecology	1	51	0	0	0	0	0	0	1.554	16.6	1	1	1	0	1	tri-weekly TC	1	1077.22	22	1
211844	breast	1	54	0	0	0	0	0	0	1.458	23.8	1	0	0	0	0	wPTX+HER	0	946.50	35	1

219361	gynecology	1	56	0	0	0	0	0	0	1.547	22.3	1	0	0	0	0	tri-weekly TC	1	1077.68	23	1
225149	breast	1	42	1	0	0	0	0	0	1.518	22.0	1	0	0	0	0	wPTX+HER	0	948.62	77	1
230131	breast	1	66	0	0	0	0	0	0	1.642	25.0	1	0	0	0	0	wPTX	0	957.37	40	0
243878	gynecology	1	66	0	0	0	0	0	0	1.452	21.6	1	0	1	1	0	tri-weekly TC	1	1078.51	48	0
257449	gynecology	1	73	0	0	0	0	0	0	1.542	16.9	1	1	1	1	0	tri-weekly TC	1	990.27	29	1
269492	gynecology	1	77	0	0	0	0	0	0	1.53	25.9	1	0	1	0	0	tri-weekly TC	1	1033.33	16	0
270123	gynecology	1	48	0	0	0	0	0	0	1.528	23.2	1	0	0	0	0	tri-weekly TC	1	1079.34	55	0
276847	breast	1	61	0	0	0	0	0	0	1.542	18.5	1	1	0	1	0	wPTX	0	910.51	49	0
277538	gynecology	1	61	0	0	1	0	0	0	1.679	25.8	1	1	1	0	1	weekly TC	1	178.51	51	1
279551	gynecology	1	44	0	0	0	0	0	0	1.593	20.9	1	0	0	0	0	tri-weekly TC	1	539.13	21	0
279690	breast	1	60	0	0	0	0	0	0	1.623	24.0	0	0	0	0	0	wPTX+HER	0	924.21	1448	1
280200	gynecology	1	69	0	0	0	0	1	1	1.234	18.4	1	1	0	0	0	tri-weekly TC	1	539.73	10	0
289290	gynecology	1	54	0	0	0	0	0	0	1.307	15.1	0	1	0	0	0	tri-weekly TC	1	1755.35	507	1
291953	breast	1	59	0	0	0	0	0	0	1.571	22.4	1	0	0	0	1	wPTX	0	916.61	49	1
293063	gynecology	1	65	0	0	0	0	1	1	1.502	19.0	1	1	1	0	1	tri-weekly TC	1	1024.09	31	1
305846	gynecology	1	60	0	0	0	0	0	0	1.617	19.8	1	1	0	0	0	tri-weekly TC	1	1078.63	0	1
309955	breast	1	66	0	0	0	0	0	1	1.433	20.4	1	0	1	0	0	wPTX	0	921.14	42	0
312060	breast	1	69	0	0	0	0	0	0	1.587	26.8	1	0	0	0	0	ddPTX	0	693.13	14	0
315361	gynecology	1	51	0	0	0	0	0	1	1.524	23.6	1	0	0	1	1	tri-weekly TC	1	1078.74	20	1
318702	breast	0	56	0	0	0	0	0	0	1.465	17.9	1	0	0	0	0	wPTX	0	1967.24	59	1
321046	breast	1	41	0	0	0	0	0	0	1.419	19.7	0	0	0	0	0	wPTX+HER	0	155.04	616	1
324314	breast	1	36	0	0	0	0	0	0	1.476	18.5	0	0	0	0	0	wPTX	0	853.66	168	0
330750	breast	1	58	0	0	0	0	0	0	1.221	15.9	1	0	0	0	0	wPTX+HER	0	933.66	21	1
339121	breast	1	72	0	0	0	0	0	0	1.434	21.4	1	0	0	0	0	wPTX+HER	0	920.50	49	1
342846	gynecology	1	44	0	0	0	0	0	0	1.867	26.4	1	0	0	0	0	tri-weekly TC	1	539.90	50	0
348556	breast	1	65	0	0	0	0	0	0	1.825	31.7	1	0	0	0	1	wPTX+HER	0	960.00	14	1
351516	breast	1	58	0	0	0	0	0	0	1.303	19.7	1	1	0	0	1	wPTX	0	920.95	28	1
356000	gynecology	1	72	0	0	0	0	0	0	1.325	15.5	1	0	0	1	1	tri-weekly TC+BEV	1	538.87	41	0
358242	breast	1	49	0	0	0	0	0	0	1.622	27.0	1	0	0	0	0	wPTX	0	924.78	42	0
366751	gynecology	1	47	0	0	0	0	0	1	1.704	20.0	1	1	0	0	0	tri-weekly TC	1	1077.46	23	0
368702	gynecology	1	70	0	0	0	0	0	0	1.444	16.9	0	0	0	0	0	tri-weekly TC	1	360.11	167	0
372047	gynecology	1	34	0	0	0	0	0	0	1.788	20.9	0	0	0	0	0	weekly TC	1	297.94	1439	1
387876	breast	1	66	0	0	0	0	0	0	1.331	21.0	1	0	0	0	0	wPTX	0	960.00	36	0
388186	gynecology	1	67	0	0	0	0	0	0	1.442	20.2	1	0	1	0	1	tri-weekly TC	1	359.21	77	0
391651	breast	1	48	0	0	0	0	0	0	1.967	31.3	1	0	0	0	0	ddPTX	0	691.41	14	1
393525	gynecology	1	51	0	0	0	0	0	1	1.524	23.6	1	0	0	1	1	tri-weekly TC	1	1078.74	20	0
394952	breast	1	63	0	0	0	1	0	0	2.121	38.6	1	0	0	0	0	wPTX+HER	0	461.57	21	0
395783	breast	1	69	0	0	0	0	0	0	1.667	25.6	1	0	0	0	0	wPTX	0	957.41	14	1
396706	gynecology	1	46	0	0	0	0	0	0	1.631	19.1	0	0	0	0	0	tri-weekly TC	1	359.17	72	1

402149	breast	1	56	0	0	0	0	0	0	1.53	24.4	1	0	0	0	0	ddPTX	0	692.81	42	0
404819	gynecology	1	66	0	0	0	0	0	0	1.626	18.7	0	0	0	0	0	tri-weekly TC	1	1077.92	418	1
407665	breast	1	55	0	1	0	0	0	0	1.623	2.6	1	0	1	0	1	ddPTX	0	690.08	42	1
408127	breast	0	73	0	0	0	0	0	0	1.485	21.1	1	0	0	1	0	wPTX+BEV	0	89.56	7	1
412006	breast	1	37	0	0	0	0	0	0	1.393	18.8	0	0	0	0	0	wPTX+BEV	0	1346.02	217	1
412289	gynecology	1	51	0	1	1	0	0	0	1.472	21.1	1	0	0	0	0	tri-weekly TC	1	668.48	24	0
418929	breast	1	54	0	0	1	0	0	0	1.478	22.9	1	0	0	0	0	ddPTX	0	690.12	15	0
423293	gynecology	1	47	0	0	0	0	0	0	1.738	25.0	0	0	0	1	0	tri-weekly TC	1	897.58	1239	0
430415	gynecology	1	67	0	0	0	0	0	1	1.576	27.7	1	1	1	0	0	tri-weekly TC	1	1077.41	61	1
433587	breast	1	55	0	0	0	0	0	0	1.61	18.4	1	0	0	0	0	ddPTX	0	695.65	14	0
438041	gynecology	1	50	0	0	0	0	0	0	1.585	23.1	1	1	1	0	0	tri-weekly TC	1	1033.30	43	0
444415	gynecology	1	61	0	0	0	0	0	0	1.6	24.0	1	1	0	0	0	tri-weekly TC	1	1080.00	24	1
446874	breast	1	70	0	0	0	0	0	0	1.209	14.3	0	0	0	0	0	wPTX	0	158.81	223	1
447678	breast	0	51	0	0	0	0	0	0	1.763	25.8	1	0	0	1	0	wPTX+BEV	0	3315.94	34	1
448718	breast	1	56	0	0	0	0	0	0	1.508	17.2	1	0	0	0	0	wPTX+HER	0	915.12	82	0
450882	breast	1	59	0	0	0	0	0	0	1.524	23.5	1	0	0	0	1	wPTX	0	944.88	7	0
453200	gynecology	0	68	0	0	0	0	1	1	1.454	24.3	1	0	0	0	0	tri-weekly TC+BEV	1	1077.03	117	0
456563	gynecology	1	47	0	0	0	0	0	1	1.704	20.0	1	1	0	0	0	tri-weekly TC	1	1077.46	23	1
458330	gynecology	1	50	0	0	0	0	0	0	1.585	23.1	1	1	1	0	0	tri-weekly TC	1	1033.30	43	1
458598	breast	1	70	0	1	0	1	0	0	1.563	23.2	1	0	0	0	0	wPTX	0	959.69	15	1
458669	breast	1	49	0	0	0	0	0	0	1.676	22.8	1	0	0	0	0	ddPTX	0	692.12	42	0
461389	gynecology	1	58	0	0	0	0	0	0	1.469	23.2	0	0	0	0	0	ddTC	1	1197.67	805	0
467204	breast	1	46	0	0	0	0	0	0	1.659	24.2	1	0	0	1	1	wPTX+HER	0	940.33	7	1
470072	breast	1	58	0	0	0	0	0	0	1.494	19.9	1	0	0	0	0	wPTX+HER	0	923.69	49	0
472173	gynecology	1	67	0	0	0	0	0	0	1.473	22.4	1	0	0	0	0	tri-weekly TC	1	1072.18	35	1
478886	breast	1	46	0	0	0	0	0	0	1.584	21.1	1	0	0	0	1	ddPTX	0	173.61	21	1
483311	breast	1	50	0	0	0	0	0	0	1.67	24.2	1	0	0	0	0	wPTX+BEV	0	4311.38	105	1
484699	gynecology	1	63	0	0	0	0	0	1	1.497	18.1	1	1	1	1	0	tri-weekly TC	1	1078.16	41	0
485957	breast	1	57	0	0	0	0	0	0	1.515	22.4	1	0	0	0	0	wPTX	0	958.42	63	1
488558	breast	1	62	0	0	0	0	0	0	1.526	24.1	1	1	1	1	1	wPTX	0	943.64	49	0
491650	gynecology	1	74	0	0	0	0	0	0	1.527	17.5	1	0	0	0	0	tri-weekly TC	1	1795.54	192	1
492338	breast	1	50	0	0	0	0	0	0	1.6	22.3	1	0	0	0	0	wPTX	0	937.50	56	0
493705	breast	1	65	0	0	0	0	0	0	1.561	23.9	1	0	1	0	0	wPTX	0	960.00	70	0
496506	breast	1	47	0	1	0	0	0	0	1.455	24.1	1	0	0	0	0	wPTX+HER	0	955.02	7	1
496909	breast	1	61	0	0	0	0	0	0	1.493	22.1	0	0	0	0	0	wPTX	0	924.31	1072	1
498715	breast	1	61	0	0	0	0	0	0	1.779	28.3	1	0	0	1	1	wPTX	0	944.35	98	1
499047	gynecology	1	61	0	0	0	0	0	1	1.503	24.8	1	1	0	0	0	tri-weekly TC+BEV	1	1062.76	44	1
501226	breast	1	46	0	0	0	0	0	0	1.475	20.6	1	0	0	0	1	wPTX+HER	0	935.59	42	0
501368	breast	1	57	0	0	0	0	0	0	1.561	28.5	1	0	0	0	0	wPTX	0	953.24	28	1

517765	breast	1	36	0	0	0	0	0	0	1.633	20.6	1	0	1	0	1	wPTX+HER	0	955.30	28	1
518379	breast	1	70	0	0	0	1	1	0	1.463	22.7	1	0	0	0	1	ddPTX	0	697.20	21	0
518717	gynecology	1	58	0	0	0	0	0	0	1.661	34.0	1	1	1	0	0	tri-weekly TC	1	928.40	36	0
523550	gynecology	1	36	0	0	0	0	0	1	1.401	20.0	1	0	0	0	0	tri-weekly TC	1	1069.23	516	1
524014	breast	1	69	0	0	0	0	0	0	1.486	30.3	1	0	0	0	0	wPTX+HER	0	952.89	63	1
535084	gynecology	0	69	0	1	0	0	0	1	1.482	26.4	1	1	0	0	1	weekly TC	1	478.07	72	1
538534	breast	1	64	0	1	0	0	0	0	1.552	23.8	1	1	1	0	1	wPTX+HER	0	960.00	84	0
545032	gynecology	1	30	1	0	1	0	0	0	1.471	18.0	1	1	0	0	0	tri-weekly TC	1	1078.05	118	1
547221	breast	1	76	0	0	0	0	0	1	1.49	24.9	1	0	0	0	1	wPTX	0	822.15	7	1
551936	breast	1	59	0	0	0	0	0	0	1.611	21.3	1	0	1	1	1	wPTX+HER	0	931.10	21	1
562998	gynecology	1	72	0	0	0	0	0	0	1.407	18.6	0	0	0	0	0	tri-weekly TC	1	1066.76	1027	1
567434	breast	1	53	0	1	0	0	0	0	1.489	20.8	1	0	0	0	0	wPTX+HER	0	886.50	77	0
567839	breast	1	53	0	0	0	0	0	0	1.729	20.8	1	0	0	0	0	wPTX	0	936.96	0	1
568547	breast	1	45	0	0	0	0	0	0	1.759	27.9	1	0	0	0	0	wPTX	0	955.09	7	1
571359	gynecology	1	58	0	0	0	0	0	0	1.647	23.2	1	1	1	0	0	tri-weekly TC	1	1078.68	42	1
571922	breast	0	68	0	0	0	0	0	0	1.383	18.3	0	0	0	0	0	wPTX	0	960.00	189	1
572168	breast	1	45	0	0	0	0	0	0	1.499	19.8	1	0	0	0	0	wPTX	0	960.00	70	0
573666	breast	1	41	0	0	0	0	0	0	1.571	21.8	1	0	0	0	0	ddPTX	0	687.46	28	0
579286	breast	1	70	0	0	1	0	0	0	1.674	24.8	1	0	0	0	0	wPTX	0	788.53	7	0
579528	gynecology	1	44	0	0	0	0	0	0	1.528	20.8	1	1	1	1	1	tri-weekly TC	1	1115.18	28	1
583985	gynecology	1	65	0	0	0	0	0	0	1.535	22.3	1	0	0	0	0	tri-weekly TC	1	1077.49	20	1
585156	breast	1	73	0	0	0	0	0	1	1.483	23.4	1	1	1	1	1	wPTX	0	2915.71	84	1
585366	breast	0	44	0	0	0	0	0	0	1.6	24.2	1	0	0	0	0	HP+PTX	0	800.00	49	1
585397	breast	1	53	0	0	0	0	0	0	1.573	25.2	0	0	0	0	0	ddPTX	0	874.13	720	1
586071	breast	1	52	0	0	0	0	0	0	1.409	17.6	0	0	0	0	0	HP+PTX	0	397.44	34	1
587235	gynecology	1	67	0	0	0	0	0	0	1.316	21.6	1	0	0	0	0	tri-weekly TC+BEV	1	1047.85	104	1
588224	gynecology	1	53	0	0	0	0	0	0	1.619	18.8	1	1	1	0	0	tri-weekly TC+BEV	1	359.48	20	0
588394	breast	1	61	0	0	0	0	0	0	1.718	26.3	1	0	0	0	0	wPTX+HER	0	960.00	14	1
592040	breast	1	46	0	0	0	0	0	0	1.44	18.2	0	0	0	0	0	wPTX+HER	0	958.33	389	0
612466	gynecology	1	48	0	0	0	0	0	0	1.676	26.4	1	0	0	0	0	tri-weekly TC	1	1076.71	108	1
615491	breast	1	62	0	0	0	0	0	0	1.366	18.4	1	0	0	0	0	wPTX+HER	0	922.40	21	0
616379	breast	1	55	0	0	0	0	1	0	1.6	21.9	0	0	0	0	0	wPTX+HER	0	937.50	1366	0
623237	breast	1	54	0	0	0	0	0	0	1.572	21.3	0	0	0	0	0	ddPTX	0	699.75	683	1
627449	breast	1	67	0	0	0	0	0	0	1.563	26.4	1	0	1	0	0	wPTX+HER	0	959.69	57	0
627688	gynecology	1	49	0	0	0	0	0	0	1.419	20.7	1	0	0	0	0	tri-weekly TC+BEV	1	1052.47	113	0
628221	gynecology	1	49	0	1	1	0	0	0	1.651	26.5	1	0	0	0	0	tri-weekly TC	1	1079.35	84	0
629589	gynecology	1	50	0	0	0	0	0	1	1.375	17.9	1	1	0	0	0	tri-weekly TC	1	1078.93	61	0
631234	gynecology	1	31	1	0	0	0	0	0	1.471	18.0	1	1	0	0	0	tri-weekly TC	1	1078.05	105	1
634950	gynecology	1	70	0	0	0	1	1	0	1.615	25.9	1	1	1	0	0	tri-weekly TC	1	1046.59	13	1

640091	gynecology	1	69	0	0	0	0	0	0	1.405	18.2	1	0	0	0	0	tri-weekly TC	1	1077.82	41	1
641521	breast	0	55	0	0	0	0	0	1	1.55	16.0	1	0	0	0	0	wPTX+BEV	0	1219.35	123	1
659196	gynecology	1	67	0	0	0	0	0	0	1.352	19.0	1	1	1	1	1	tri-weekly TC	1	1078.40	0	1
659734	gynecology	1	73	0	0	0	0	0	1	1.952	35.9	1	1	0	0	1	tri-weekly TC	1	1077.42	21	1
661420	breast	1	65	0	0	0	0	0	1	1.564	20.8	1	1	0	0	1	wPTX	0	920.89	21	0
666741	breast	1	56	0	0	0	0	0	0	1.589	26.0	1	0	0	0	0	wPTX+HER	0	943.99	56	0
667156	breast	1	48	0	0	0	0	0	0	1.464	23.5	1	0	0	0	1	wPTX	0	778.69	28	1
680046	breast	1	47	0	0	0	0	0	0	1.494	23.8	1	0	0	0	1	wPTX+HER	0	960.00	63	1
680822	gynecology	0	61	0	0	0	0	0	0	1.394	23.1	0	0	0	0	0	tri-weekly TC	1	358.68	46	1
690046	gynecology	1	68	0	0	0	0	0	1	1.482	26.9	1	1	1	0	0	tri-weekly TC	1	1069.36	34	1
690164	breast	1	54	0	1	0	0	0	0	1.658	25.9	1	0	1	0	1	wPTX	0	940.89	70	0
690308	breast	1	66	0	0	0	0	0	0	1.328	17.4	0	0	0	0	0	wPTX+HER	0	948.80	1120	0
692773	gynecology	1	68	0	0	0	0	0	1	1.526	19.5	1	1	0	0	0	tri-weekly TC	1	673.21	34	0
693328	gynecology	1	57	0	0	0	0	0	1	1.586	24.7	1	0	1	0	1	tri-weekly TC	1	1008.26	30	1
699203	breast	1	54	0	0	0	0	0	0	1.659	24.3	0	0	0	0	0	wPTX+HER	0	650.99	98	0
700544	breast	1	73	0	0	0	0	0	0	1.25	20.6	0	0	0	0	0	HP+PTX	0	240.00	22	1
700994	gynecology	1	76	0	0	0	0	0	0	1.519	29.1	1	1	1	1	0	tri-weekly TC	1	719.10	35	0
702421	gynecology	1	69	0	0	0	0	0	0	1.468	14.7	1	0	0	1	0	TP	1	784.74	1	1
702779	breast	1	70	0	0	0	0	0	1	1.575	30.0	1	0	0	0	0	wPTX+HER	0	720.00	49	0
705515	gynecology	1	47	1	0	1	0	0	0	1.585	17.8	1	1	0	0	0	tri-weekly TC	1	1078.86	80	0
705666	breast	1	65	0	0	0	1	0	0	1.622	23.6	1	0	0	0	0	wPTX+BEV	0	625.77	35	0
710145	breast	1	73	0	0	0	0	0	0	1.682	25.4	1	0	0	0	0	wPTX+HER	0	927.47	14	1
712761	gynecology	1	47	0	1	1	0	1	1	1.986	32.9	1	0	0	0	0	tri-weekly TC	1	1078.55	64	1
714326	breast	1	69	0	1	1	0	0	0	1.417	18.1	1	0	1	0	0	wPTX	0	931.55	35	0
720870	breast	1	78	0	0	0	0	1	0	1.507	23.7	1	0	0	0	0	wPTX+HER	0	955.54	49	0
722018	breast	1	58	0	0	0	0	0	0	1.64	21.9	1	0	0	0	0	wPTX+HER	0	713.41	7	0
723918	breast	1	61	0	0	0	0	0	1	1.455	20.7	1	0	0	0	0	wPTX	0	956.70	56	0
726715	gynecology	1	76	0	0	0	0	0	0	1.324	19.6	1	1	0	1	0	tri-weekly TC	1	1078.55	44	1
742358	breast	1	73	0	0	0	0	0	0	1.245	20.1	1	0	0	0	0	wPTX+HER	0	838.68	21	0
743004	gynecology	1	53	0	0	0	0	0	0	1.478	19.1	1	0	0	0	0	tri-weekly TC	1	1079.84	43	1
754122	gynecology	1	47	0	0	0	0	0	0	1.714	26.8	1	1	1	0	0	tri-weekly TC	1	359.39	21	1
755166	gynecology	1	55	0	1	0	0	0	0	1.525	23.4	1	0	1	1	0	tri-weekly TC	1	1078.03	23	0
759061	breast	1	66	0	0	0	0	0	0	1.508	19.4	1	0	0	0	0	wPTX+HER	0	954.91	28	0
762693	breast	1	39	0	0	0	0	0	0	1.686	24.7	1	0	0	0	0	ddPTX	0	699.88	1113	1
763785	gynecology	1	64	0	0	0	0	0	0	1.563	25.6	1	1	0	1	1	tri-weekly TC	1	1033.91	44	1
767424	breast	1	43	0	0	0	0	0	0	1.604	21.0	1	0	0	0	0	wPTX+HER	0	960.00	18	0
768230	breast	1	71	0	0	0	0	1	0	1.482	20.1	1	0	1	1	1	wPTX+HER	0	931.17	48	0
780707	breast	1	57	0	0	0	0	0	0	1.601	19.3	1	0	0	0	0	ddPTX	0	699.56	56	0
783146	breast	1	57	0	1	0	0	0	0	1.566	19.4	1	0	0	0	0	ddPTX	0	700.00	46	0

783509	gynecology	1	59	0	0	0	0	0	0	1.644	26.0	1	1	1	0	0	tri-weekly TC	1	538.32	56	0
787779	gynecology	1	62	0	0	1	0	0	0	1.572	23.9	1	1	1	0	0	tri-weekly TC	1	1076.59	14	1
795239	breast	0	65	0	0	0	0	0	1	1.594	27.1	1	0	0	0	0	wPTX	0	398.37	7	1
800112	gynecology	1	50	1	0	0	0	0	0	1.512	24.2	1	0	0	0	0	tri-weekly TC	1	1079.37	95	0
805397	breast	0	60	0	0	0	0	0	0	1.32	19.9	1	0	0	0	0	wPTX	0	727.27	14	1
807180	gynecology	1	38	0	0	0	0	0	0	1.566	22.5	1	0	0	0	0	tri-weekly TC	1	1076.63	21	0
809048	breast	1	32	0	0	0	0	0	0	1.503	22.7	1	0	0	0	1	wPTX+HER	0	958.08	28	1
810282	gynecology	1	70	0	0	0	0	0	1	1.363	23.7	1	1	1	0	1	tri-weekly TC+BEV	1	1402.08	28	1
810990	breast	1	38	0	0	0	0	0	0	1.453	17.8	0	0	0	0	0	ddPTX	0	585.00	1259	1
819187	breast	1	51	0	0	0	0	0	0	1.415	21.7	0	0	0	0	0	wPTX	0	399.29	49	1
820970	gynecology	1	76	0	0	0	0	0	1	1.519	29.1	1	1	1	1	0	tri-weekly TC	1	719.10	35	0
826078	breast	1	39	0	0	0	0	0	0	1.583	19.8	0	0	0	0	0	wPTX	0	355.75	300	0
827310	breast	1	47	0	0	0	0	0	0	1.464	18.8	0	0	0	0	0	wPTX	0	901.64	322	1
827893	breast	1	77	0	0	0	0	0	0	1.58	23.8	1	0	0	0	0	wPTX+HER	0	899.37	42	0
832669	breast	1	71	0	0	0	0	0	0	1.347	17.3	1	0	0	1	1	wPTX	0	935.41	84	1
833367	gynecology	1	45	0	0	0	0	0	0	1.484	16.9	1	0	1	1	0	tri-weekly TC	1	1078.80	81	1
838485	breast	1	63	0	0	0	0	0	0	1.422	19.8	1	0	0	0	0	wPTX+BEV	0	535.86	14	1
844526	breast	1	65	0	0	0	0	0	0	1.288	17.7	0	0	0	0	1	wPTX+HER	0	931.68	1413	0
847406	gynecology	0	40	0	0	1	0	0	0	1.379	14.5	1	0	0	0	0	tri-weekly TC	1	354.28	4	1
852689	breast	1	43	0	0	0	0	0	0	1.685	19.0	1	0	0	0	0	wPTX+HER	0	940.89	21	1
857800	breast	1	50	0	0	0	0	0	1	1.638	24.5	1	0	0	0	1	wPTX	0	952.38	49	1
859151	gynecology	1	34	0	0	0	0	0	0	1.522	19.8	1	0	0	0	0	tri-weekly TC	1	1077.32	101	0
860581	gynecology	1	47	0	0	0	0	0	0	1.419	18.2	1	1	0	1	0	tri-weekly TC	1	1077.84	122	0
861745	gynecology	1	73	0	0	0	0	0	0	1.509	20.8	1	0	0	0	0	tri-weekly TC	1	1078.24	105	1
863483	breast	1	61	0	1	1	0	0	0	1.429	20.5	1	0	0	0	0	wPTX+HER	0	839.75	135	0
866270	breast	1	70	0	0	0	0	0	1	1.595	24.1	1	0	1	0	0	wPTX	0	902.82	28	0
867315	gynecology	1	54	0	1	0	0	0	0	1.696	27.5	1	0	0	0	1	tri-weekly TC	1	1077.35	12	1
871489	breast	1	71	0	0	0	0	0	0	1.474	18.3	1	0	0	0	1	wPTX	0	468.11	98	1
871671	breast	1	58	0	0	0	0	0	0	1.726	25.8	1	0	0	0	1	wPTX+HER	0	938.59	84	0
883494	breast	1	74	0	0	0	0	0	0	1.396	20.7	1	0	0	0	1	wPTX	0	945.56	35	1
886672	gynecology	1	55	0	0	0	0	0	0	1.598	24.4	1	1	1	0	0	tri-weekly TC	1	1077.60	77	1
887160	gynecology	1	76	0	0	0	1	0	1	1.498	22.2	1	1	1	0	0	tri-weekly TC	1	674.66	53	0
905413	breast	1	35	0	0	0	0	0	0	1.673	28.5	1	0	0	0	0	wPTX	0	953.97	35	1
918844	breast	1	73	0	0	0	0	0	1	1.346	20.0	1	1	1	0	0	ddPTX	0	698.37	28	0
924434	breast	0	59	0	0	0	0	0	0	1.518	18.7	1	1	0	0	1	wPTX+BEV	0	2144.27	56	1
926305	breast	1	45	0	0	0	0	0	0	1.597	27.9	1	0	0	0	0	wPTX	0	864.12	28	1
928975	gynecology	1	58	0	0	0	0	1	1	1.936	32.1	1	1	0	0	0	tri-weekly TC	1	985.55	14	0
930039	breast	1	73	0	0	0	0	0	0	1.542	20.5	1	0	0	0	0	wPTX	0	856.03	84	0
932069	breast	1	47	0	0	0	0	0	0	1.357	19.1	1	0	0	0	0	wPTX+HER	0	928.52	63	0

932185	breast	1	46	0	0	0	0	0	0	1.732	23.9	1	0	0	0	0	wPTX+HER	0	956.12	56	0
934462	breast	1	54	0	0	0	0	0	0	1.568	20.9	0	0	0	0	0	wPTX	0	159.49	49	0
938483	breast	1	52	0	0	0	1	0	0	1.703	27.1	1	0	0	0	1	wPTX	0	958.31	7	0
951912	gynecology	1	31	1	0	0	0	0	0	1.471	18.0	1	1	0	0	0	tri-weekly TC	1	1078.05	105	0
961140	breast	1	55	0	1	0	0	0	0	1.457	20.5	1	0	0	0	0	wPTX+HER	0	947.15	91	0
963116	breast	1	46	0	0	0	0	0	0	1.591	27.5	1	0	0	0	0	wPTX+HER	0	957.89	70	1
965631	gynecology	1	78	0	0	0	0	0	0	1.576	21.1	1	1	0	0	1	tri-weekly TC	1	897.44	92	0
965908	breast	1	66	0	1	0	0	0	0	1.475	17.8	1	0	0	0	0	ddPTX	0	691.53	14	1
969938	breast	1	66	0	0	0	0	0	1	1.504	25.2	1	1	0	0	1	ddPTX	0	658.24	14	1
972999	breast	1	48	0	0	0	0	0	0	1.519	19.7	1	0	0	0	0	ddPTX	0	697.83	14	0
975232	breast	1	72	0	0	0	0	0	0	1.475	18.7	1	0	0	0	0	wPTX	0	935.59	42	0
976981	gynecology	1	59	0	0	0	0	0	0	1.914	35.9	1	1	0	1	0	tri-weekly TC	1	987.18	9	0
977720	gynecology	1	41	0	0	0	0	0	0	1.593	20.9	1	0	0	0	0	tri-weekly TC	1	939.27	94	0
979303	breast	1	61	0	0	0	1	0	1	1.791	34.8	1	0	0	0	1	wPTX+HER	0	859.85	14	0
981448	breast	1	59	0	0	0	0	0	0	1.486	22.5	1	0	0	0	1	wPTX+HER	0	952.89	31	1
