# Supplementary material for: Risk factors and pharmacotherapy for chemotherapy-induced peripheral neuropathy in paclitaxel-treated female cancer survivors: A retrospective study in Japan
Source: PLoS One. 2021 Dec 31;16(12):e0261473. doi: 10.1371/journal.pone.0261473 (PMC8719717; doi:10.1371/journal.pone.0261473)
Supplement: S1 Table — PCT, paclitaxel; ddPCT, dose-dense paclitaxel; wPCT, weekly paclitaxel; HP, trastuzumab + pertuzumab; BEV, bevacizumab; HER, trastuzumab; ddTC, dose-dense paclitaxel + carboplatin; TP, paclitaxel + cisplatin; tri-weekly TC, tri-weekly paclitaxel + carboplatin; wTC, weekly paclitaxel + carboplatin. (PDF) [file pone.0261473.s004.pdf]

S1 Table. Chemotherapy regimens used in female breast and gynecologic cancer patients treated with paclitaxel at Kindai University Hospital

Breast cancer (n = 162)		Gynecologic cancers (n = 121)	
Regimen	n	Regimen	n
ddPCT	24	ddTC	1
wPCT	65	TP	1
wPCT + HP	3	tri-weekly TC	104
wPCT + BEV	10	tri-weekly TC + BEV	11
wPCT + HER	60	wTC	4

PCT, paclitaxel; ddPCT, dose-dense paclitaxel; wPCT, weekly paclitaxel; HP, trastuzumab + pertuzumab; BEV, bevacizumab; HER, trastuzumab; ddTC, dose-dense paclitaxel + carboplatin; TP, paclitaxel + cisplatin; tri-weekly TC, tri-weekly paclitaxel + carboplatin; wTC, weekly paclitaxel + carboplatin.
